# The Inlay Technique in Alveolar Ridge Augmentation: A Systematic Review

**DOI:** 10.3390/jcm14051684

**Published:** 2025-03-02

**Authors:** Carlo Barausse, Subhi Tayeb, Gerardo Pellegrino, Lorenzo Bonifazi, Edoardo Mancuso, Stefano Ratti, Andrea Galvani, Roberto Pistilli, Pietro Felice

**Affiliations:** 1 Oral Surgery Unit, Department of Biomedical and Neuromotor Sciences (DIBINEM), University of Bologna, 40125 Bologna, Italy; subhi.tayeb@studio.unibo.it (S.T.); gerardo.pellegrino2@unibo.it (G.P.); lorenzo.bonifazi2@unibo.it (L.B.); pietro.felice@unibo.it (P.F.); 2 Cellular Signaling Laboratory, Anatomy Center, Department of Biomedical and Neuromotor Sciences (DIBINEM), University of Bologna, 40126 Bologna, Italy; stefano.ratti@unibo.it; 3 Prosthodontic Unit, Department of Biomedical and Neuromotor Sciences (DIBINEM), University of Bologna, 40125 Bologna, Italy; edoardo.mancuso@unibo.it; 4 Department of Biomolecular Sciences (DISB) University of Urbino, 61029 Urbino, Italy; andrea.galvani6@unibo.it; 5 Unit of Oral and Maxillofacial Surgery, San Camillo-Forlanini Hospital, 00152 Rome, Italy; r_pistilli@libero.it

**Keywords:** bone regeneration, implant dentistry, implant survival, inlay technique, interpositional grafting, review, sandwich technique, systematic review, vertical ridge augmentation

## Abstract

**Background/Objectives**: Vertical ridge augmentation remains a critical challenge in implant dentistry for addressing inadequate alveolar bone height. The inlay technique, or sandwich osteotomy, has gained attention for its potential to improve graft vascularization and predictability. This systematic review aimed to evaluate the clinical outcomes of the inlay technique. **Methods**: A systematic search was conducted in Cochrane Library and Medline databases for studies published from 2015 to 2025 to capture the most recent studies and advancements specifically focusing on the inlay technique. Inclusion criteria encompassed observational and interventional studies, including randomized controlled trials (RCTs) and cohort and case series with a focus on outcomes related to the inlay technique. Key outcomes were extracted and analyzed, including implant survival rates, MBL, vertical bone gain, and surgical complications. **Results**: Eleven studies involving 352 patients and more than 612 implants were included, with a mean follow-up of 2.27 ± 2.69 years (range: 4 months to 8 years). The implant survival rates ranged from 84.5% to 100%. Mean vertical bone gain varied from 2.69 to 4.4 mm. Complications were fewer with the inlay technique compared to onlay and other grafting methods, with significantly reduced graft-related failures and soft tissue issues. **Conclusions**: The inlay technique shows good vertical bone augmentation with high implant survival rates and fewer complications compared to other reconstructive techniques. Longer follow-up studies are needed to support its value in managing vertically deficient ridges. Moreover, further studies with extended follow-up are required to evaluate long-term marginal bone loss.

## 1. Introduction

Vertical ridge augmentation is a fundamental procedure in implant dentistry, addressing the challenges posed by inadequate alveolar bone height for successful implant placement [1,2,3]. Various surgical techniques including onlay grafting, guided bone regeneration (GBR), and distraction osteogenesis have been developed to compensate for insufficient vertical bone volume [4,5]. Onlay grafts place the graft material on the external cortical plate and often provide robust volumetric gains, but they can be prone to flap dehiscence and graft exposure due to the extensive soft tissue manipulation required [6,7]. GBR, meanwhile, typically uses barrier membranes to contain particulate grafts; although effective, it demands careful flap advancement and may involve lengthy healing intervals, especially in severe vertical defects [8,9,10]. Distraction osteogenesis is another alternative but entails specialized equipment, longer treatment time, and substantial patient compliance. Despite the efficacy of these approaches, they can present limitations such as prolonged healing, higher morbidity, and unpredictable outcomes, particularly in anatomically challenging cases [11,12,13]. An alternative option, when feasible and adequate bone height remains, is the placement of short implants, which may reduce the need for extensive grafting and capitalize on the residual native bone [14]. However, short implants may not be viable in situations where significant vertical dimensions are required or in high-esthetic areas.

The inlay or “sandwich” technique has emerged as a promising alternative for vertical ridge augmentation. Originally described by Schettler in 1974 [15] for augmenting atrophic mandibles and later adapted by Sailer in 1989 [16] for maxillary reconstructions, this approach leverages an intrabony environment to reduce graft exposure and foster enhanced vascularization. By utilizing interpositional bone grafts between mobilized bone segments, this method enhances vascularization and promotes graft integration. Despite some advantages, the inlay technique requires advanced surgical skills and meticulous planning to minimize complications, such as graft resorption, infection, and implant failure. Compared to onlay procedures, inlay grafts rely on the mobilized segment’s inherent blood supply, potentially allowing for more predictable bone formation and decreased resorption over time.

A wide range of grafting materials can be used with the inlay technique, including autogenous bone (for instance, harvested from the iliac crest or mandibular ramus), allogeneic blocks, xenografts (bovine or equine), and particulate bone substitutes [17]. Autogenous grafts typically have excellent osteogenic potential but demand a second surgical site, increasing morbidity. Allogeneic and xenogeneic materials avoid donor-site morbidity, yet they may exhibit variable handling properties and integration rates. Regardless of the graft source, meticulous segment fixation is essential to prevent micromotion and optimize graft consolidation, especially when significant vertical gains (ranging from 4 to 10 mm) are pursued.

A growing body of literature has explored the efficacy of the inlay technique in terms of implant survival rates, vertical bone gain, and complications. However, significant variability exists in the reported outcomes, influenced by differences in study designs, patient populations, and follow-up durations [18]. Moreover, advancements in digital workflows and patient-specific guides have introduced new dimensions to this technique, further warranting a comprehensive evaluation of its current clinical relevance.

This review aims to critically analyze the available evidence on the inlay technique for vertical ridge augmentation, focusing on key parameters such as implant survival rates, marginal bone loss, vertical bone gain, and complications. By synthesizing data from randomized controlled trials and clinical studies, this review seeks to provide a thorough understanding of the technique’s clinical utility and identify areas for future research to optimize patient outcomes.

## 2. Materials and Methods

This systematic review was conducted to evaluate the applications and outcomes of the inlay technique, also known as the sandwich or interpositional technique, in alveolar ridge augmentation. The review adheres to applicable elements of the Preferred Reporting Items for Systematic Reviews and Meta-Analyses (PRISMA) guidelines to ensure transparency and methodological rigor [19].

### 2.1. Search Strategy

A systematic literature search was performed in two independent resources: PubMed, for accessing MEDLINE and other indexed databases, and the Cochrane Library, for systematic reviews and clinical trials. The search was performed on 1 January 2025, covering the period from 1 January 2015 to 1 January 2025. The search strategy combined the following terms:

*((inlay [tiab] OR sandwich [tiab] OR interpositional [tiab])AND (technique [tiab] OR approach [tiab] OR procedure [tiab] OR graft [tiab] OR)) AND (oral [tiab] OR alveolar [tiab] OR maxillofacial [tiab] OR dental [tiab] OR “Oral Surgical Procedures” [MeSH])*

Reference lists of selected articles were manually screened to identify additional relevant studies.

### 2.2. Inclusion and Exclusion Criteria

Studies were included if they were published between 2015 and 2025 and addressed the clinical application of the inlay technique for alveolar ridge augmentation. Only primary research studies were considered, including randomized controlled trials (RCTs), split-mouth RCTs, and case series with a minimum of five cases, all focusing on practical and clinical outcomes. The inclusion criteria also specified studies involving human participants aged 18 years or older, with data on specific anatomical regions such as the posterior mandible and anterior maxilla. To ensure a focus on primary research, systematic reviews, narrative reviews, and meta-analyses were excluded. Studies were excluded if they involved animal models, in vitro experiments, or lacked full-text availability. Only articles written in English were considered. Duplicate articles identified across databases were removed.

### 2.3. Article Selection Process

The initial search identified a total of 29 articles. This limited number reflects the highly specific focus of the review on the inlay technique for vertical ridge augmentation, as well as the strict inclusion criteria applied to ensure relevance and quality. After removing 2 duplicates using EndNote 21 Software (Clarivate, Philadelphia, PA, USA), 27 studies remained. Titles and abstracts were screened based on the inclusion and exclusion criteria, resulting in the exclusion of 9 articles due to irrelevance. Of the remaining 18 studies, 2 were excluded because full texts were unavailable despite contacting the authors and allowing a 14-day response period. Three additional articles were excluded after a full-text review as they involved the same patient cohorts as other included randomized controlled trials but reported shorter follow-up periods. One study was excluded after the full-text review as it focused exclusively on histological outcomes. Ultimately, 12 studies were included in the final analysis. The article screening was conducted independently by two oral surgeons and implantologists with expertise in research. In cases of disagreement, the opinion of a third reviewer with a similar background was sought to reach a consensus. A PRISMA flow diagram was created to illustrate the selection process.

### 2.4. Data Extraction and Quality Assessment

**Figure 1:**
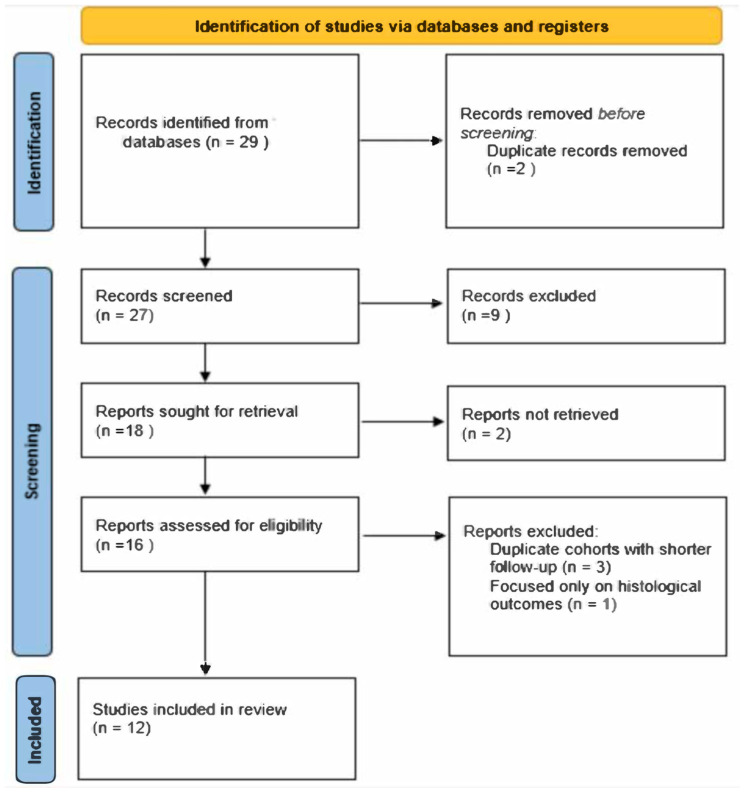
This diagram illustrates the systematic process of identifying, screening, and selecting studies for inclusion in the systematic review.

**Figure 2:**
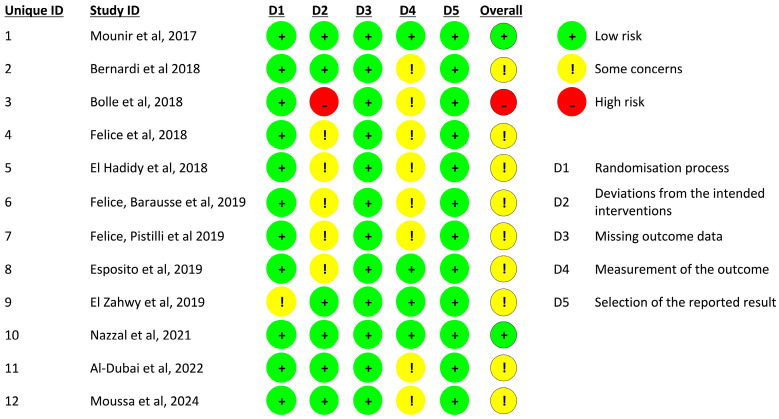
This table presents the assessment of the risk of bias for each included study, evaluated across the five domains of the RoB 2 tool [21,22,23,24,25,26,27,28,29,30,31,32].

For each study, efforts were made to extract key clinical parameters, including the implant survival rate, complication rates associated with the surgical technique, marginal bone loss (MBL), and the vertical bone gain achieved (i.e., the height of the augmented ridge). Data extraction was conducted systematically to ensure consistency across studies. The risk of bias was assessed using the Cochrane Risk of Bias 2 (RoB 2) tool [20], focusing on five domains: bias arising from the randomization process, bias due to deviations from intended interventions, bias due to missing outcome data, bias in the measurement of the outcome, and bias in the selection of the reported result. The evaluation was conducted independently by two experienced reviewers, and any disagreements were resolved through discussion or, when necessary, consultation with a third reviewer (Figure 1 and Figure 2).

## 3. Results

A total of 12 studies were included in this systematic review, involving 352 patients and more than 612 implants. The studies encompassed randomized controlled trials, split-mouth trials, and multicenter designs. The follow-up period ranged from a minimum of 4 months to a maximum of 5 years, with a mean follow-up of 2.28 ± 2.69 years. The studies assessed various parameters including implant survival rates, marginal bone loss (MBL), vertical bone gain, and complications associated with the inlay technique.

### 3.1. Implant Survival Rate

The implant survival rates reported across the included studies showed variations based on the augmentation technique, implant length, and follow-up duration.

In the study by Al-Dubai et al. (2022) [21], a 100% survival rate 4 months after placement was observed for implants placed using the sandwich osteotomy technique, irrespective of whether the implant placement was simultaneous or delayed.

In the trial by Moussa et al. (2024) [22], which compared digital workflows to conventional sandwich osteotomy techniques, the survival rates for implants placed in both groups were not explicitly reported but were noted to be high, with no major implant failures observed during the study period.

Different studies compared the inlay technique with short implants placed in native bone. In the randomized controlled trial conducted by Bernardi et al. (2018) [23], survival rates were compared between short implants (6 mm) and standard-length implants (10 mm) placed in augmented mandibular sites. The 6 mm implants achieved a survival rate of 94.2%, outperforming the 10 mm implants, which showed an 84.5% survival rate. However, this difference was not statistically significant (*p* = 0.57)

Similarly, the multicenter trial by Bolle et al. (2018) [24] compared survival rates of 4 mm short implants and >10 mm implants placed in augmented bone. The implant survival rates were comparable between the groups, with no significant difference reported (*p* = 1.0).

In the study by Esposito et al. (2019) [25], which investigated 5 mm implants with a calcium-incorporated titanium surface versus >10 mm implants in augmented sites, implant survival rates were also found to be similar between groups, with no significant difference (*p* = 0.609).

Felice, Pistilli et al. (2019) [26] (6 mm× 4 mm vs. ≥10 mm implants at 5 years) recorded four short-implant failures (two in the maxilla and two in the mandible) and three augmented-site prostheses not placed (one associated with the loss of three implants). The authors found no significant difference in overall implant survival between short and longer implants (*p* = 1.00).

In another, Felice, Barausse et al. (2019) [27] trial (5 mm vs. ≥10 mm implants at 5 years), three short implants and one long implant failed in the mandible, while three short implants and one long implant failed in the maxilla. Again, there was no statistically significant difference in implant failure rates between the two groups (*p* = 1.00).

Lastly, Felice et al. (2018) [28] (6.6 mm vs. ≥9.6 mm implants at 8 years) reported five short implants failing in three patients vs. three long implants failing in three other patients; no difference in implant survival was detected (*p* = 1.00).

### 3.2. Vertical Bone Gain

The included studies reported varying degrees of vertical ridge augmentation, reflecting the influence of operator skills, technique, materials, and patient factors.

In the study by Al-Dubai et al. (2022) [21], vertical ridge augmentation was assessed using the sandwich osteotomy technique with simultaneous and delayed implant placement in the anterior maxilla. The reported mean vertical bone gain was 4.04 ± 0.59 mm in the simultaneous placement group and 3.86 ± 0.52 mm in the delayed placement group. The difference between the two groups was not statistically significant (*p* = 0.518), indicating that both approaches provide comparable vertical augmentation outcomes.

The study by Nazzal et al. (2021) [29] introduced a computer-guided sandwich osteotomy technique and compared it to the conventional approach. The mean vertical bone gain in the computer-guided group was 4.4 mm, slightly higher than the 3.9 mm observed in the conventional group. While both techniques yielded similar vertical gains, the computer-guided method demonstrated a significant reduction in palatal tipping (*p* < 0.001), suggesting improved precision and predictability in the guided approach.

Moussa et al. (2024) [22] compared a digital workflow to a conventional sandwich osteotomy technique in a split-mouth study involving vertically deficient posterior mandibles. The study reported a statistically significant higher vertical bone gain in the digital workflow group, with a mean of 3.76 ± 0.72 mm compared to 2.69 ± 0.37 mm in the conventional group (*p* = 0.001). The authors attributed the improved outcomes in the digital workflow group to enhanced accuracy in osteotomy execution and fixation of the transport segment.

El Hadidy et al. (2018) [30] examined the buccal and palatal approaches for segmental sandwich osteotomy in the anterior maxilla. Both techniques resulted in significant vertical ridge augmentation, with a mean postoperative vertical bone gain percentage of 79.9% for the buccal approach and 76.5% for the palatal approach. The differences were not statistically significant (*p* = 0.43), indicating the efficacy of both approaches for vertical bone regeneration in the aesthetic zone.

El Zahwy et al. (2019) [31] compared onlay and inlay grafting techniques with simultaneous implant placement in the anterior maxilla. The inlay technique (sandwich osteotomy) achieved a significantly higher vertical bone gain of 3.34 mm compared to −0.02 mm in the onlay group (*p* < 0.05). The negative value in the onlay group was attributed to graft resorption and wound breakdown, which were not observed in the inlay group. The authors concluded that the sandwich technique is superior for vertical ridge augmentation in this context.

In a study by Mounir et al. (2017) [32], vertical ridge augmentation using onlay grafting with titanium mesh was compared to the sandwich technique. The sandwich group achieved a higher mean percentage of vertical bone gain (31.6%) compared to the onlay group (20.7%), although the difference was not statistically significant (*p* = 0.2). The authors noted that both techniques were effective but highlighted the technical complexity and potential for graft exposure associated with the onlay approach.

### 3.3. Complications

The studies reviewed reported various complications associated with vertical ridge augmentation techniques, ranging from mild soft tissue issues to graft failures and infections.

In the study by Al-Dubai et al. (2022) [21], complications were minimal, with one case of a cracked bony segment in the control group during fixation screw placement. This complication was managed successfully with an emergency screw and particulate bone graft, and it did not affect the final outcome.

Nazzal et al. (2021) [29], in their pilot study comparing computer-guided and conventional sandwich osteotomy techniques, reported one case of a cracked bony segment in the computer-guided group. Despite this complication, the final outcomes were not affected.

Moussa et al. (2024) [22] observed one case of soft tissue dehiscence in their split-mouth study comparing digital workflow and conventional sandwich osteotomy techniques. The case was handled immediately by debridement, followed by watertight closure: the wound healed uneventfully thereafter. The study highlighted that a digital workflow could potentially reduce the incidence of complications by improving procedural accuracy.

In the study conducted by El Hadidy et al. (2018) [30], no significant complications were reported in either the buccal or palatal approach groups. Both techniques showed uneventful wound healing, reflecting the relative safety of these approaches in anterior maxillary vertical ridge augmentation.

El Zahwy et al. (2019) [31] reported a total of 12 distinct complications in the onlay group: one patient experienced a cracked graft block during implant insertion, three had flap dehiscence at 10 days and two at 1 month. Graft mobility was observed in three patients, leading to the failure of three implants, while two others had complete graft loss, and one patient showed implant thread exposure. Notably, some of these complications may have overlapped in the same individuals. In contrast, no complications were reported in the inlay group.

M. Mounir et al. (2017) [32] observed a single patient that experienced flap dehiscence and mesh exposure approximately 10 days postoperatively in the onlay group. The mesh remained exposed for some time, requiring daily irrigation. Healing then occurred by secondary intention. No complications occurred in the inlay group.

Regarding the studies comparing the inlay technique with short implants, Bernardi et al. (2018) [23] reported a high rate of complications in augmented sites compared to sites treated with 6 mm implants. Specifically, 22 out of 36 patients experienced complications in the augmented sites, and the most common complication was paraesthesia.

Bolle et al. (2018) [24] reported a higher incidence of complications among patients who underwent mandibular bone augmentation than among those treated with 4 mm short implants. Specifically, 9 augmented patients out of 20 (in the mandible) presented complications compared with only 1 in the short implant group (*p* = 0.003).

Similarly, Esposito et al. (2019) [25] at 5-year follow-up reported that in the mandible, two augmented patients were not rehabilitated due to multiple complications, and three implants (including one replacement) failed in a single augmented patient; by contrast, only one short implant (and its crown) was lost at 2 years. Significantly more complications occurred in augmented mandibles (17 augmented patients vs. 9 with short implants, difference in proportion = 0.39; 95% CI: 0.10 to 0.62; *p* = 0.013). Reported complications included graft failures, multiple implant losses, and delayed or absent prosthetic rehabilitation.

Felice, Pistilli et al. (2019) [26] compared 6 × 4 mm implants placed in posterior atrophic jaws against longer implants (≥10 mm) in augmented bone (5-year post-loading). They found 19 complications among 14 augmented patients versus 5 among 4 short-implant patients, a difference that was not statistically significant (*p* = 0.118).

In another study, Felice, Barausse et al. (2019) [27] assessed 5 mm implants in posterior atrophic jaws against longer implants in augmented sites (also 5-year data). They observed 16 complications in 11 short-implant patients vs. 14 in 12 long-implant patients (no significant difference, *p* = 1.00).

Finally, Felice et al. (2018) [28] compared 6.6 mm short implants to vertically augmented sites in posterior mandibles (8-year follow-up). They reported 27 complications in 22 augmented patients vs. 9 in 8 short-implant patients, and this time, the difference was significant (*p* < 0.001).

### 3.4. Marginal Bone Loss (MBL)

Regarding marginal bone loss (MBL), few studies reported specific data, given the short period of follow-up.

El Zahwy et al. (2019) [31] directly compared onlay and inlay grafting techniques in the anterior maxilla but did not study the marginal bone loss around implants. However, the inlay group exhibited significantly less crestal bone loss (1.65 mm vs. 4.77 mm, *p* < 0.05).

Bernardi et al. (2018) [23] reported no significant differences in MBL between 6 mm and 10 mm implants placed in augmented mandibles.

Bolle et al. (2018) [24] provided more detailed data on MBL, particularly in mandibles. At 4 months post-loading, patients with mandibular short implants lost on average 0.40 mm of peri-implant bone at 4 months and patients with 10 mm or longer mandibular implants lost 0.52 mm. This difference was found statistically significant (*p* = 0.006).

Esposito et al. (2019) [25] extended the analysis of MBL to include 5 × 5 mm implants with a nanostructured calcium-incorporated titanium surface. Patients with mandibular short implants lost on average 1.22 mm of peri-implant bone at 5 years and patients with 10 mm or longer mandibular implants lost 1.70 mm (*p* = 0.004).

Felice, Pistilli et al. (2019) [26] compared 6 mm × 4 mm implants vs. ≥10 mm implants at 5 years. In the mandible, short implants had a mean bone loss of 1.34 ± 0.35 mm vs. 2.11 ± 0.59 mm in the augmented group, a significant difference of 0.77 mm (*p* = 0.003).

Another Felice, Barausse et al. (2019) [27] study assessed 5 mm implants vs. longer implants: after 5 years, mandibular bone loss averaged 1.72 mm with short implants vs. 2.10 mm around longer implants (*p* = 0.022).

Lastly, Felice et al. (2018) [28] (8-year follow-up) reported that 6.6 mm implants lost an average of 1.58 mm vs. 2.46 mm for longer implants in augmented sites—a significant difference of 0.88 mm in favor of the short implants (Table 1).

### 3.5. Study Limitation

This systematic review has several limitations that warrant discussion. First, the included studies exhibit heterogeneity in terms of sample size, follow-up duration, patient selection criteria, grafting materials, and surgical protocols, which complicates direct comparisons and reduces the ability to perform meta-analyses. Second, the limited number of high-quality RCTs reduces the overall strength of evidence. Third, the lack of standardized methods for measuring key outcomes, such as vertical bone gain and marginal bone loss (MBL), introduces potential measurement bias and limits the generalizability of the findings. Additionally, the relatively short follow-up periods in many studies prevent a comprehensive evaluation of long-term outcomes and stability of the augmented bone.

Another significant bias arises from the fact that several of the included studies were conducted by the authors of this review. This overlap could introduce an element of author bias, despite efforts to maintain objectivity during the critical appraisal process. Finally, publication bias cannot be excluded, as studies with favorable outcomes are more likely to be published, potentially skewing the overall conclusions.

## 4. Discussion

This systematic review analyzed 12 studies investigating the inlay (sandwich) technique for vertical ridge augmentation, focusing on implant survival rates, marginal bone loss (MBL), vertical bone gain, and complications.

The inlay osteotomy technique has emerged as a promising approach to addressing vertical alveolar bone deficiencies, offering clinicians a potentially more predictable alternative to other augmentation methods. Indications for the inlay technique include cases of vertical alveolar ridge deficiencies with sufficient horizontal bone volume to stabilize the mobilized segment, as well as situations where predictable vascularization of the grafted segment is a priority. This technique is particularly suitable for the posterior mandible with 4–6 mm of residual bone height above the mandibular canal and for the anterior maxilla, where aesthetic outcomes are paramount. Conversely, contraindications include patients with inadequate horizontal bone volume, significant systemic conditions that impair bone healing, or poor oral hygiene, which increases the risk of infection and graft failure. Achieving successful outcomes relies on accurate diagnosis, careful patient selection, and informed clinical decision-making, underscoring the importance of thorough preoperative planning and precise surgical execution [33,34].

Across multiple studies, vertical bone gains have ranged between approximately 2.7 mm and 4.4 mm, and these improvements often reached statistical significance (e.g., *p* < 0.05) when compared to conventional onlay techniques or other grafting modalities. Importantly, the inlay approach appears to provide enhanced vascularization for the grafted segment, as the bone block or particulate graft is interposed between viable bony walls, thereby reducing the risk of resorption and potentially leading to more stable outcomes [35,36]. In many of the trials considered, implant survival rates were reported above 90%, further underlining the procedure’s reliability [19,20,21].

Several investigations compared this technique to the placement of short implants in native bone in the posterior regions. While short implants showed similarly high survival rates and reduced morbidity—as exemplified by studies reporting over 90% success rates with no statistically significant difference from standard-length implants (*p* > 0.05)—they may pose aesthetic and prosthetic drawbacks. Due to the limited crown height necessitated by shorter fixtures, the final prosthesis can appear disproportionate or less natural. In contrast, the inlay technique facilitates vertical ridge reconstitution, enabling the placement of standard-length implants that maintain ideal crown-to-implant ratios. This dimensional preservation is especially critical in the aesthetic zone, where an inadequate implant length or suboptimal angulation can compromise soft tissue contours and papillary support. Consequently, although short implants can significantly reduce operative time and postoperative complications in the posterior mandible and maxilla, their universal applicability is limited.

Nevertheless, the inlay osteotomy technique is not without its challenges. Precise and stable repositioning of the mobilized segment is crucial; even minor deviations during the surgical phase can lead to complications such as segment fractures or poor flap adaptation. In some studies, these intraoperative complications were managed successfully with immediate fixation or grafting without impacting implant success, but they nonetheless highlight the technique’s steep learning curve. The use of patient-specific digital workflows has been shown to mitigate these risks; in one trial, the digitally guided group reported significantly fewer bony fractures and reduced palatal tipping of the mobilized segment (*p* < 0.001). Similarly, computer-aided design (CAD) allowed more precise flap design and a more accurate three-dimensional reapproximation of the segment, further limiting soft tissue complications such as flap dehiscence.

Another point of debate in the literature is the long-term stability of regenerated bone. While certain authors documented stable vertical augmentation for up to five years [26,27], others noted gradual crestal resorption beyond the first year of loading, which could ultimately reduce the effective vertical gain achieved [31]. Importantly, longer follow-up times in excess of eight years have revealed higher rates of complications, graft resorption, and marginal bone loss in augmented sites, sometimes reaching statistical significance compared with short implants (*p* < 0.05) [29]. However, these findings may also be influenced by operator experience, varying graft materials, and differences in patient selection [37,38,39,40,41,42]. Indeed, precise protocols for measuring vertical bone gain—often using standardized radiographs or three-dimensional scans—remain inconsistent across studies, limiting definitive comparisons [43].

In light of these considerations, certain recommendations for future research emerge [44,45,46,47]. First, large-scale randomized controlled trials (RCTs) with harmonized protocols for radiographic measurements (e.g., standardized cone-beam computed tomography) are needed to more reliably quantify vertical gains. Second, separating the outcomes of anterior versus posterior sites may further elucidate the relative benefits of inlay osteotomy vis-à-vis short implants, particularly concerning aesthetic considerations. Finally, cost–benefit analyses and patient-reported outcome measures (PROMs)—which remain underreported—would provide valuable insights into overall patient satisfaction, quality of life, and resource allocation.

## 5. Conclusions

In summary, current evidence supports the inlay osteotomy technique as an effective and predictable method for achieving vertical ridge augmentation with high implant survival rates, particularly when aesthetics is a primary concern. While short implants remain an excellent solution in situations where residual native bone suffices—often with fewer complications—they may not consistently yield the ideal tissue contours or prosthetic outcomes. The incorporation of digital workflows offers a promising avenue for reducing complication rates and enhancing the precision of this challenging procedure. Over the long term, continued research with standardized methodologies and extended follow-up is required to substantiate these findings, refine selection criteria, and optimize patient outcomes.

## Figures and Tables

**Table 1:** This table provides an overview of the studies selected for inclusion in the systematic review. It outlines key information for each study.

Title	Year	Authors	Study Design	Participants	Follow-Up	Comparison	Implant Survival Rate	MBL at Follow-Up	Complications	Average Vertical Bone Gain
Assessment of vertical ridge augmentation in anterior aesthetic zone using onlay xenografts with titanium mesh versus the inlay bone grafting technique: A randomized clinical trial	2017	Mounir et al. [32]	RCT	16 patients, 40 implants	6 months postoperative	Onlay vs. inlay bone grafting	Not reported	Not reported	1 dehiscence in the onlay group	20.7% (onlay), 31.6% (inlay). No difference between groups (*p* = 0.2)
Short Versus Longer Implants in Mandibular Alveolar Ridge Augmented Using Osteogenic Distraction: One-Year Follow-up of a Randomized Split-Mouth Trial	2018	Bernardi et al. [23]	Split-mouth RCT	36 patients, 170 implants	1 year post-loading	6 mm vs. 10 mm implants	94.2% for 6 mm implants, 84.5% for 10 mm implants (*p* = 0.5705)	Similar in both groups	22/36 patients with complications in augmented sites. Higher at augmented sites (*p* < 0.05)	Not reported
4 mm long vs. longer implants in augmented bone in posterior atrophic jaws: 1-year post-loading results from a multicentre randomised controlled trial	2018	Bolle et al. [24]	Multicenter RCT	80 patients (40 in mandible)	1 year post-loading	4 mm vs. >10 mm implants in augmented sites	No difference in implant failure (*p* = 1)	Less bone loss with short implants in mandible (0.40 mm vs. 0.52 mm) (*p* = 0.006)	9/20 complications in augmented sites and fewer complications with short implants (*p* = 0.003)	Not reported
Short implants versus longer implants in vertically augmented posterior mandibles: result at 8 years after loading from a randomised controlled trial	2018	Felice et al. [28]	RCT	60 patients	8 years	6.6 mm long implants vs. 9.6 mm or longer implants in augmented sites	5 failures in short implants vs. 3 failures in augmented sites (*p* = 1)	Short implant: 1.58 mm MBL compared with 2.46 mm in the augmented group	More complications in augmented patients (27 in 22 augmented patients vs. 9 in 8 patients of the short implant group) (*p* < 0.001)	Not reported
Assessment of vertical ridge augmentation and labial prominence using buccal versus palatal approaches for maxillary segmental sandwich osteotomy (inlay technique): A randomized clinical trial	2018	El Hadidy et al. [30]	RCT	16 patients, 40 implants	4 months postoperative	Buccal vs. palatal approach	Not reported	Not reported	No complications	79.9% (buccal), 76.5% (palatal). No significant difference (*p* = 0.43)
Five-year results from a randomised controlled trial comparing prostheses supported by 5-mm long implants or by longer implants in augmented bone in posterior atrophic edentulous jaws	2019	Felice, Barausse et al. [27]	RCT (split-mouth)	30 patients (15 in mandible and 15 in maxilla)—56 implants	5 years post-loading	5 mm vs. >10 mm implants in augmented sites	No difference in implant failure (*p* = 1)	Short implants: 1.72 mm MBL compared with 2.10 mm in the augmented group (*p* = 0.022)	12 patients with 14 complications in augmented sites. No difference compared with short implants	Not reported
Posterior atrophic jaws rehabilitated with prostheses supported by 6-mm-long 4-mm-wide implants or by longer implants in augmented bone. Five-year post-loading results from a within-person randomised controlled trial	2019	Felice, Pistilli et al. [26]	Within-person RCT	40 patients (20 in mandible and 20 in maxilla)—88 implants	5 years post-loading	6 × 4 mm vs. >10 mm implants in augmented sites	No difference in implant failure (*p* = 1)	Short implants: 1.34 ± 0.35 mm MBL compared with 2.11 ± 0.59 mm in the augmented group (*p* = 0.003)	14 patients with 19 complications in augmented sites. No difference compared with short implants (*p* = 0.118)	Not reported
Posterior atrophic jaws rehabilitated with prostheses supported by 5 × 5 mm implants with a nanostructured calcium-incorporated titanium surface or by longer implants in augmented bone. Five-year results from a randomised controlled trial	2019	Esposito et al. [25]	RCT	80 patients (40 in mandible)—63 implants	5 years post-loading	5 × 5 mm vs. >10 mm implants in augmented sites	No difference in implant failure (*p* = 0.609)	Short implant: 1.22 mm MBL compared with 1.70 mm in the augmented group (*p* = 0.004)	More complications in augmented site compared to short implant in mandible (0.013)	Not reported
Assessment of vertical ridge augmentation and marginal bone loss using autogenous onlay vs. inlay grafting techniques with simultaneous implant placement in the anterior maxillary esthetic zone: A randomized clinical trial	2019	El Zahwy et al. [31]	RCT	16 patients, 40 implants	6 months postoperative	Onlay vs. inlay grafting	Not reported	Crestal bone loss: 4.77 mm in onlay vs. 1.65 mm in inlay (*p* < 0.05)	Onlay group: 12 complications. Inlay group: No complications	3.34 mm (inlay), −0.02 mm (onlay) (*p* < 0.05)
Maxillary vertical alveolar ridge augmentation using computer-guided sandwich osteotomy technique with simultaneous implant placement versus conventional technique: A pilot study	2021	Nazzal et al. [29]	RCT (pilot study)	12 patients	4 months postoperative	Computer-guided vs. conventional sandwich osteotomy	Not reported	Not reported	1 complication in study group. Significantly reduced palatal tipping with computer-guided technique (*p* < 0.001)	4.4 mm (guided), 3.9 mm (conventional)
Maxillary vertical alveolar ridge augmentation using sandwich osteotomy technique with simultaneous versus delayed implant placement: A proof of principle randomized clinical trial	2022	Al-Dubai et al. [21]	RCT	16 patients, 35 implants	4 months postoperative	Simultaneous vs. delayed implant placement	100%	Not reported	1 complication in the control group	4.04 mm (simultaneous), 3.86 mm (delayed). No significant difference (*p* = 0.518)
Assessment of bone gain and neurosensory affection with the sandwich osteotomy technique for vertically deficient posterior mandible using a full digital workflow versus conventional protocol: A randomized split mouth study	2024	Moussa et al. [22]	Split-mouth RCT	10 patients, 40 implants	4 months postoperative	Digital workflow vs. conventional sandwich osteotomy	Not reported	Not reported	1 case of dehiscence (resolved)	3.76 mm (digital), 2.69 mm (conventional) (*p* = 0.001)

## Data Availability

No new data were created or analyzed in this study. Data sharing is not applicable to this article.

## Abbreviations

The following abbreviations are used in this manuscript:

MBL	Marginal bone loss
RCT	Randomized controlled trial
CI	Confidence interval
PRISMA	Preferred Reporting Items for Systematic Reviews and Meta-Analyses
CAD/CAM	Computer-aided design/computer-aided manufacturing
